# Investigating the Impact of Substrate Preheating on the Thermal Flow and Microstructure of Laser Cladding of Nickel-Based Superalloy

**DOI:** 10.3390/ma17020399

**Published:** 2024-01-12

**Authors:** Zhibo Jin, Xiangwei Kong, Liang Ma

**Affiliations:** 1 School of Mechanical Engineering and Automation, Northeastern University, Shenyang 110819, China; zhibojin@163.com (Z.J.); maliangneu@163.com (L.M.); 2 Key Laboratory of Vibration and Control of Aero-Propulsion System, Ministry of Education, Northeastern University, Shenyang 110819, China; 3 Liaoning Province Key Laboratory of Multidisciplinary Design Optimization of Complex Equipment, Northeastern University, Shenyang 110819, China

**Keywords:** laser cladding, melt pool heat transfer, dendrite spacing, substrate preheating, additive manufacturing simulation

## Abstract

The preheating of the substrate in laser additive superalloys can reduce residual stress and minimize cracking. However, this preheating process can lead to changes in the heat transfer conditions, ultimately affecting the resulting microstructure and mechanical properties. In order to explore the influence of substrate preheating on the formation of laser cladding, this research focuses on investigating the characteristics of Inconel 718, a nickel-based superalloy, as the subject of study. To simulate the temperature and flow field of laser cladding, a 3D computational fluid dynamics (CFD) model is employed. By varying the initial preheating conditions, an investigation is conducted into the distribution of the temperature field under different parameters. This leads to the acquisition of varying temperature gradients, G, and solidification speeds, R. Subsequently, an analysis is carried out on both the flow field and solidification microstructure in the melt pool. The results demonstrate that the preheating of the substrate results in a slower cooling rate, ultimately leading to the formation of a coarser microstructure.

## 1. Introduction

Substrate preheating is an important factor because it can change the thermal process of the process. Related studies have shown that preheating the substrate/workpiece before the deposition process can reduce the thermal stress generated during the deposition process and ultimately reduce/eliminate the resulting delamination and cracking [1,2,3], and it is used in multiple applications, such as joining, welding, and laser melt deposition [4,5,6]. However, the preheating effect may affect the final expected properties of the deposited material. Nickel-based superalloys have a high fracture strength and excellent high-temperature corrosion resistance. However, laser cladding technology can be used for repairing components or rapid manufacturing, greatly reducing costs [7].

The modeling and calculation of laser cladding are complex. By establishing a 3D transient numerical model, the temperature distribution and velocity distribution in the laser additive manufacturing process can be obtained [8,9,10] and the size [11,12,13], temperature distribution [14,15], and cooling rate during the process can be calculated. Raghavan et al. [16] used an improved dendritic growth model to predict the transition from columnar to equiaxed crystals. Morville et al. [17] proposed a two-dimensional thermal transport numerical model to simulate the manufacturing process of multi-layer additives. The ALE (Arbitrary Lagrangian Eulerian Method) method is used to track the gas/liquid interface in the molten pool [18,19,20,21]. Debroy et al. [22] proposed a three-dimensional transient transport numerical model to study the multi-layer laser additive manufacturing process with coaxial powder feeding. The calculation results obtained from its model are used to explain and evaluate the texture evolution of the solidification structure in the experiment. Fallah et al. [23] investigated the influence of localized surface preheating on microstructural alterations and crack formation in laser-direct-deposited samples. Corbin et al. [24] studied the effect of substrate surface preheating on part deformation in laser cladding using in-situ measurement and distortion measurement. Liu et al. [25] proposed a method of dynamic preheating and created a 3D finite element model to analyze the influence of dynamic preheating on the thermal behavior of multi-pass and multi-layer laser cladding. To guarantee the desired mechanical properties of the deposited layer under preheating conditions, it is essential to gain a deeper understanding of the heat and mass transfer behavior of the melt, as well as the solidification behavior of the deposited layer.

Therefore, in this study, a 3D CFD model is utilized to simulate the heat transportation behavior of the laser additive manufacturing process. The heat transfer characteristics between the deposited material and substrate are analyzed, and the impact of substrate preheating and double-layer overlap on thermal behavior, temperature and flow field features, and morphology of the microstructure formed are evaluated. The experimental results verified the accuracy of the model, which offers benefits in the prognostication of the microstructure of laser additive manufacturing.

## 2. Models and Parameters

The model of laser cladding is reasonably simplified, as follows [26]:

(1) The flow of liquid fluid in the molten pool is Newtonian, laminar and incompressible;
(2) The mushy region is assumed to be a porous medium with isotropic permeability;
(3) The heat flux generated by the heated powder and the heat loss caused by evaporation are disregarded;
(4) The impact of shielding gas, feeding gas and powder on the melt pool surface is omitted.

**Figure 1:**
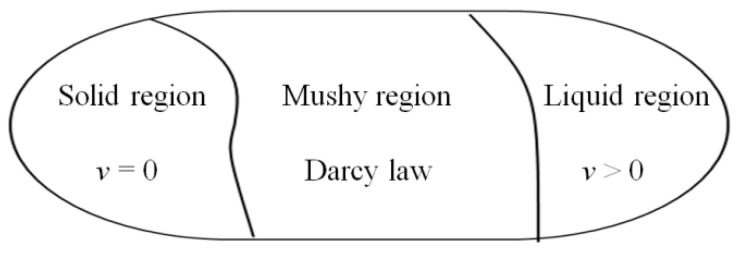
Schematic diagram of solid–liquid phase variable region.

The concept of a liquid phase volume fraction is introduced to handle the division of solid–liquid intervals in phase transition problems. The range of the solid phase region and the liquid phase region changes over time. The convection term in the energy equation of the solid domain differs from that of the fluid domain, and the momentum equation only affects the flow field. Figure 1 illustrates how the volume fraction unifies the equations of the two domains. In the solid domain, only the energy equation with the unsteady term needs to be calculated. In the liquid domain, both the energy and momentum equations with the convection term should be calculated. In the mushy region, the energy equation involving the change in latent heat of the phase transition must be calculated, along with the momentum equation that corresponds to Darcy’s law.

The continuity equation is as follows:

(1) $$\frac{\partial \rho }{\partial t}+\nabla \cdot \left(\rho u\right)=0$$

Due to the existence of the S/L phase change in the laser cladding process, the influence of latent heat of melting should be considered in the heat transport equation and the energy equation is as follows [10]:

(2) $$\rho c_{p}\left(\frac{\partial T}{\partial t}+u\cdot \nabla T\right)=\nabla \cdot \left(k\nabla T\right)-\frac{\partial H}{\partial t}-\rho u\cdot \nabla H$$

where *ρ* is density, *c_p_* is specific heat, *T* is temperature, *t* is time, *k* is heat conduction dilution, ∆*H* is melting latent enthalpy capacity; ∆*H* = *Lf_l_*, *L* is latent heat of melting. The liquid mass fraction *f_l_* is defined as

(3) $$f_{l}=\left\{\begin{array}{ll} 1, & \;\;T>T_{l}\\ \frac{T-T_{s}}{T_{l}-T_{s}}, & \;T_{s}<T\leq T_{l}\\ 0, & \;\;T<T_{s} \end{array}\right.$$

The incompressible Navier–Stokes equation is used to describe the liquid metal in the molten pool.

(4) ρ∂u∂t+ρu⋅∇u=∇⋅(μ∇u)−∂p∂x+K01−FlFl3+Bu

where ***μ*** is the viscosity, *p* is the pressure, and the third term on the right side of the Equation (4) represents the momentum dissipation term in the mushy region, which is based on the Carman–Kozeny equation [27]. *K*_0_ is a constant related to the mushy region, and *B* is a very small number to prevent the denominator from being zero, Linear interpolation is used to interpolate the thermophysical parameters of the mushy region.

The Lagrangian idea is used to solve the boundary flow situation of the free surface, and the displacement of the grid is mainly determined by two parts: one is the mass addition caused by the powder entering the free surface, and the other is the impact of the melt pool flow speed on the advancement of the liquid surface.

(5) $$V=V_{p}(t,x)+u$$

where ***u*** is the interface movement caused by fluid velocity, and *V_p_*(*t*, *x*) is the interface movement caused by the addition of powder into the free surface. According to the powder model, it is known that

(6) $$\;V_{p}\left(t,x\right)=\frac{2m_{f}\eta_{m}}{\rho_{m}\pi r_{p}^{2}}\exp\left(\frac{-2{\left(x-X\left(t\right)\right)}^{2}+y-Y{\left(t\right)}^{2}}{r_{p}^{2}}\right)$$

where *m_f_* is the powder feeding speed, *η_m_* is the powder capture rate, *ρ_m_* is the powder density, *r_p_* is the light source radius, *X*(*t*) and *Y*(*t*) are the moving trajectories of the laser head over time.

The initial temperature value is set to room temperature 300 K. All six surfaces of the cuboid include convection and radiation. The laser energy input *ql* on the upper surface is:

(7) $$q_{l}=Q(x,y,t)\eta_{p}$$

where *η_p_* is the absorption rate and *Q*(*x*, *y*, *t*) is the effective energy of the laser, which is described in Equation (8) by a mixture of two fundamental modes with 40% TEM1 and 60% TEM2. The source beam profile is a near flat-top form [23].

(8) $$Q(x,y,t)=\frac{2P}{\pi r_{l}^{2}}(a+\frac{2br^{2}}{r_{l}^{2}})e^{-2r^{2}/r_{l}^{2}}$$

where *a* is the fraction of the TEM1 model; *b* is the fraction of the TEM2 model; *P* is the heat source power; *r_l_* is the radius of the laser light source.

The principal dendrite arm spacing (PDAS) in the microstructure is based on the Kurz criterion. When the solidification speed is fast [28],

(9) $$\lambda =4.3{\left(\frac{\Delta T_{0}D\Gamma }{k_{0}}\right)}^{\frac{1}{4}}G^{-\frac{1}{2}}R^{-\frac{1}{4}}$$

where *D* is the diffusion coefficient 3 × 10^−9^ m^2^·s^−1^ and *Γ* is the Gibbs–Thomson coefficient. This calculation takes 3.65 × 10^−7^ K·m and *k*_0_ is the distribution coefficient 0.48.

The model is a cuboid of 24 mm × 16 mm × 7 mm, and the cladding surface is the upper surface of the cuboid. It is both the input boundary of the energy equation and the input boundary of the mass transfer equation, and it is also the lifting surface of the ALE dynamic grid. The laser scans in the positive direction of X. The physical properties of the Inconel 718 are shown in Table 1, and the values of basic simulation parameters are listed in Table 2. The model was implemented using the commercial finite element software COMSOL Multiphysics.

## 3. Experiment Scheme

A coaxial powder feeding laser cladding device is used, and Inconel 718 powder with a good fluidity and a particle size of 45~105 μm is selected, with a particle size distribution of D50 = 37 μm and D90 = 58 μm. The powder is dried at 100 °C for 2 h before the experiment using a drum-type dryer. The distribution of powder element content is as shown in Table 3.

**Figure 2:**
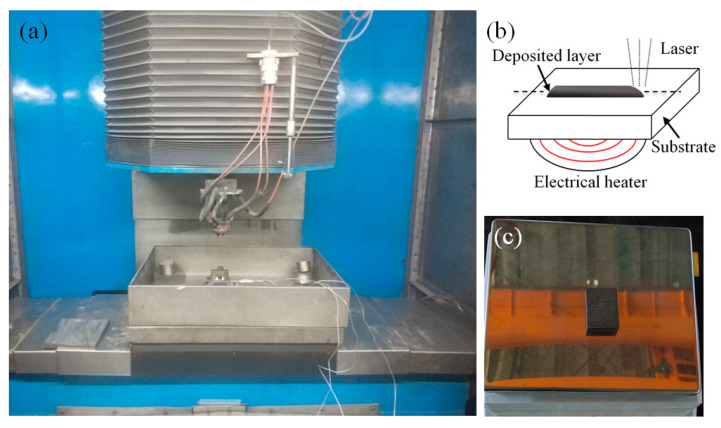
Equipment layout diagram: (**a**) cladding platform; (**b**) preheating schematic diagram; (**c**) preheating platform.

The laser source is a CO_2_ laser with a maximum power of 4000 W and a spot diameter of 1.1 mm. The equipment layout and substrate preheating diagram are shown in Figure 2. The laser cladding system comprises laser equipment, a numerical control workbench, and a powder feeder with four nozzles symmetrically distributed. Argon gas delivers the metal powder to the substrate, creating a steady powder flow and anti-oxidation environment. Figure 2a illustrates the laser cladding process. The schematic diagram and layout of electrical heating are shown in Figure 2b,c. The three preheating schemes are no preheating, 200 °C, and 400 °C, respectively.

The substrate is made of Inconel 718 and underwent annealing and stress-relieving treatment. Three cross-sections, perpendicular to the scanning direction, were wire-cut and then ground and polished successively with 240#, 600#, 800#, 1200#, and 2000# sandpaper, followed by W3.5 and W2.5 polishing pastes. The samples were then subjected to corrosion using a solution of HCl, H_2_SO_4_, and CuSO_4_ for 25 s and observed under a metallographic microscope. The metallographic microscope was used to observe the geometrical morphology and count the primary dendrite arm spacing.

## 4. Results and Discussion

### 4.1. Temperature Field and Characteristic Parameters

**Figure 3:**
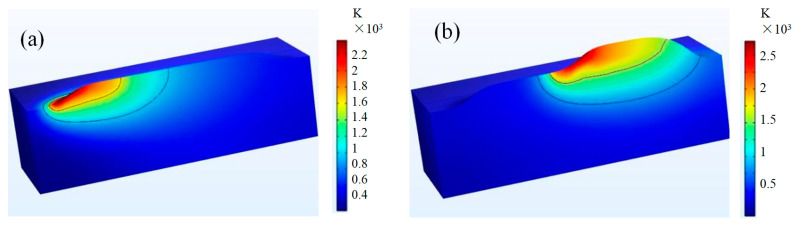
Temperature distribution at different times of double-layer cladding: (**a**) first layer temperature distribution; (**b**) second layer temperature distribution.

During laser cladding, the free boundary constantly inputs energy as the laser source moves, causing the temperature in the melt pool to gradually increase. Within 0.5 s, the temperature field of the melt pool reaches a quasi-steady state, with the isotherm shape remaining largely unaltered. Figure 3 depicts the temperature distribution cloud map of double-layer cladding without substrate preheating. The model’s high-temperature region is primarily situated in the melt pool and adjoining sample area, exhibiting a “tailing” shape with a maximum temperature above 2000 K. A substantial temperature differential is observed between the high-temperature region and the substrate, with the majority of the substrate region below 500 K. Due to heat accumulation, the melt pool in the second layer is larger than in the first layer, as demonstrated by Figure 3a,b. The melt width is 19.1% longer, and the melt depth is 112% deeper without preheating the substrate, which has a bigger effect on the melt depth. Following the melting of the first layer, the temperature in the substrate reduces more gradually in comparison to the surface. Thus, in the second layer, additional heat accumulates in the direction of the melt depth, leading to an increased depth of the molten material.

**Figure 4:**
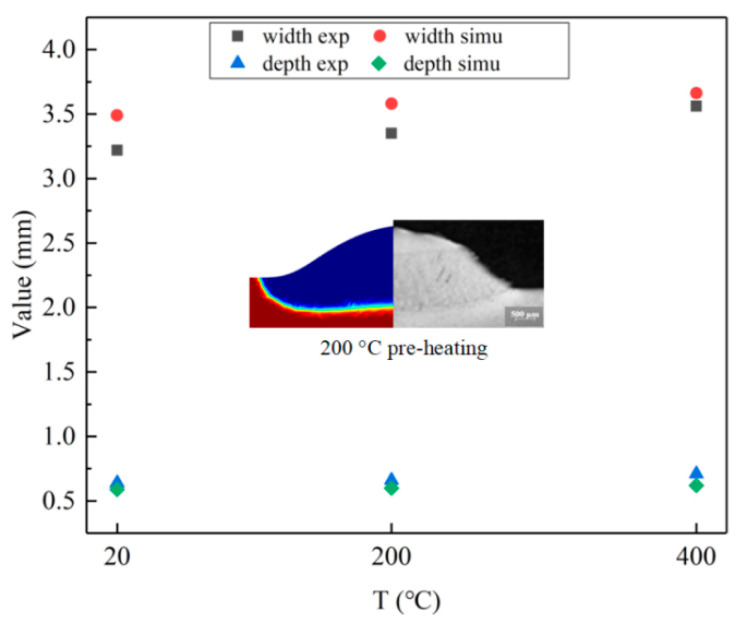
Comparison of experimental and simulated values for molten pool size.

Figure 4 shows the comparison of molten pool sizes between the simulated and experimental values under different preheating conditions. It can be seen that the maximum error of the melt pool width obtained by the simulation is 8.4%, and the maximum error of the melt pool depth is 12.7%. The errors of other points are within 10%, indicating that the simulation results are reliable. Under the same deposition parameters, the melt pool size after preheating is larger. When preheating to 400 °C, the melt pool width is 10.6% larger than that without preheating, and the melt pool depth increases by 10.9%. After preheating, the melt pool boundary is wider, so the powder in the deposition area is higher.

**Figure 5:**
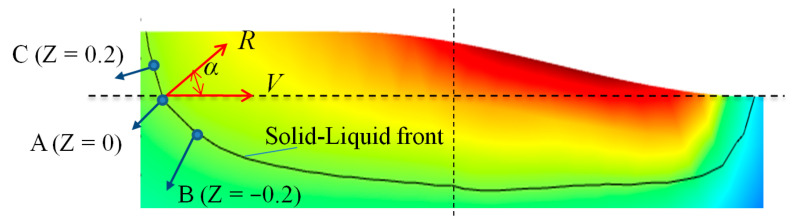
Single-layer cladding diagram.

**Figure 6:**
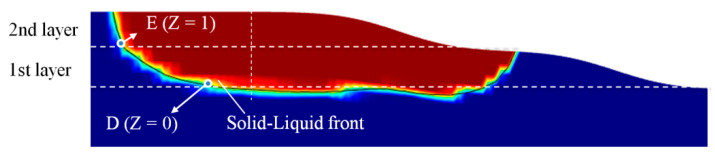
Double-layer cladding diagram.

In this paper, the symmetrical cross-section in the center of the melt pool is extracted, using the temperature of 1571 K as the isotherm. The left part of the contour line indicates the solidification interface. The single-layer and double-layer cladding processes of three kinds of preheating cladding types are simulated. For the single-layer cladding layer, three points are selected, A (Z = 0 mm), B (Z = −0.2 mm) and C (Z = 0.2 mm), as shown in Figure 5. Finally, the solidification interface is located at the rear edge of the melt pool. After reaching the quasi-steady state, the temperature gradient *G*and solidification rate *R* remains essentially constant. *α* is the angle between the laser scanning direction and solidification interface normal direction. During the double-layer cladding process, two benchmark points, D (Z = 0 mm) and E (Z = 1 mm), are chosen for each layer. This selection is based on the melt height of the first layer, which is roughly 1 mm, as depicted in Figure 6.

**Figure 7:**
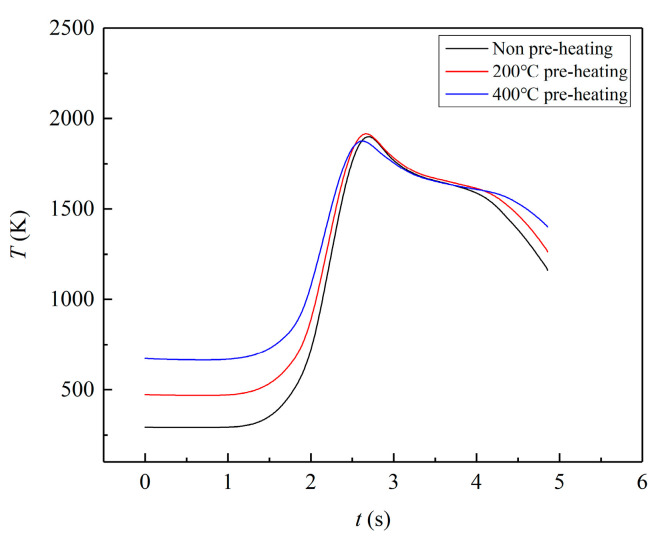
Temperature of single layer (Z = −0.2 mm).

Figure 7 depicts the temperature history graph of point B (Z = −0.2 mm) point under three preheating conditions. As the laser source moves, the temperature of point B gradually increases under the three working conditions, eventually reaching its maximum temperature of 1900 K. Although the preheating temperature of 400 °C is higher than the other two working conditions, the peak temperature is comparable, and the rate of temperature increase and decrease is slower. This results in a reduction in the temperature gradient *G* and consequently minimizes the thermal stress during the forming process.

**Figure 8:**
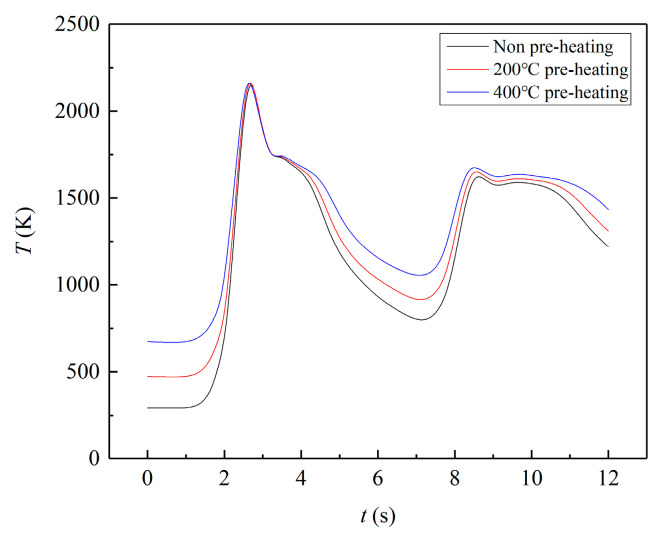
Temperature of double layer (Z = 0 mm).

Figure 8 illustrates the temperature history of three types of preheating cladding for double-layer cladding D (Z = 0 mm), and two distinct temperature peaks are visible. In all three conditions, the first temperature peak exceeds 2210 K, and the temperature difference is less than 10 K. However, the second temperature peaks, with maximum temperatures of 1621 K, 1648 K and 1669 K, respectively, exceed the melting point, indicating that the region has undergone two melting and solidification processes. The figure reveals that as the temperature increases, the relative temperature difference between the three conditions decreases. Conversely, when the temperature decreases, the relative temperature difference enlarges.

**Figure 9:**
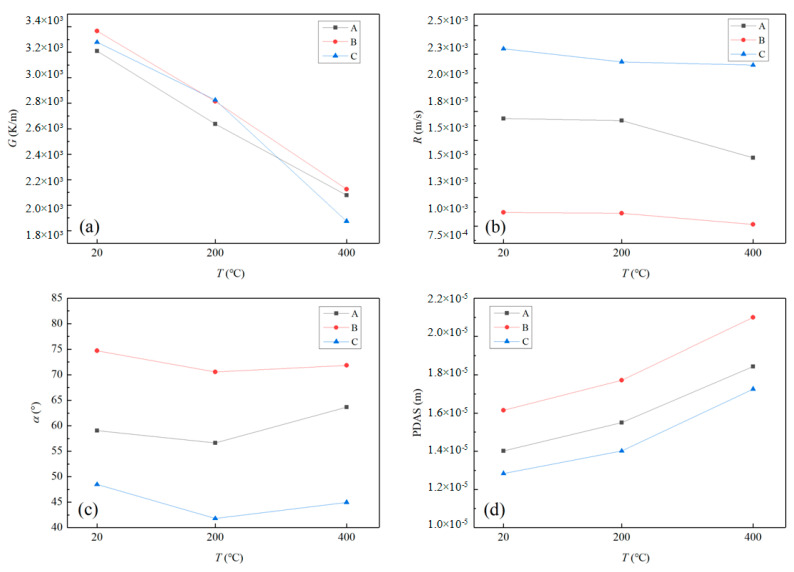
Comparison of parameter values for three preheating conditions of single-layer cladding: (**a**) temperature gradient; (**b**) solidification rate; (**c**) solidification angle; (**d**) PDAS.

By comparing the parameter values for points A (Z = 0 mm), B (Z = −0.2 mm), and C (Z = 0.2 mm) displayed in Figure 9, it is evident that an increase in preheating temperature leads to a significant linear reduction in the temperature gradient G at every point, as depicted in Figure 9a. This is due to the fact that substrate preheating raises the temperature of the substrate, which reduces the temperature difference between the cladding layer and the substrate, ultimately leading to a significant reduction in *G* during the process. It should be noted that point C is located closer to the upper surface of the fusion layer, which makes it more vulnerable to factors such as the thickness of the fusion layer and surface heat dissipation. When the preheating temperature is set to 400 °C, the powder absorption rate increases, which in turn leads to an increase in the thickness of the fusion layer. However, this can also cause significant changes in the solute flow within the melt, resulting in a lower temperature gradient value at point C.

When preheating the substrate to 400 °C, the *G*-value of point A decreases by 37%, and the solidification speed *R* decreases by 20% as shown in Figure 9b. Meanwhile, the solidification speeds of points B and C decrease by less than 10%. This indicates that multiple factors affect solidification speed, which is not a linear relationship. The angle *α* is determined by the laser scanning direction and the normal direction, with preheating showing no significant influence, as shown in Figure 9c. The combination parameter, cooling rate (*G* × *R*), has a direct effect on the size of the microstructure, whereby greater cooling rates lead to smaller microstructures. Conversely, the morphological parameter (*G*/*R*) influences the morphology of the microstructure. We calculated the PDAS values for different processes using Formula (5), as shown in Figure 9d. It has been observed that an increase in the cladding layer results in a higher cooling rate, leading to a smaller PDAS. As the preheating temperature rises, the cooling rate decreases accordingly. If point A point is preheated to 400 °C, the PDAS value is 131% of its unpreheated state. Hence, elevating the preheating temperature can mitigate thermal stress and also lead to a coarser microstructure.

**Figure 10:**
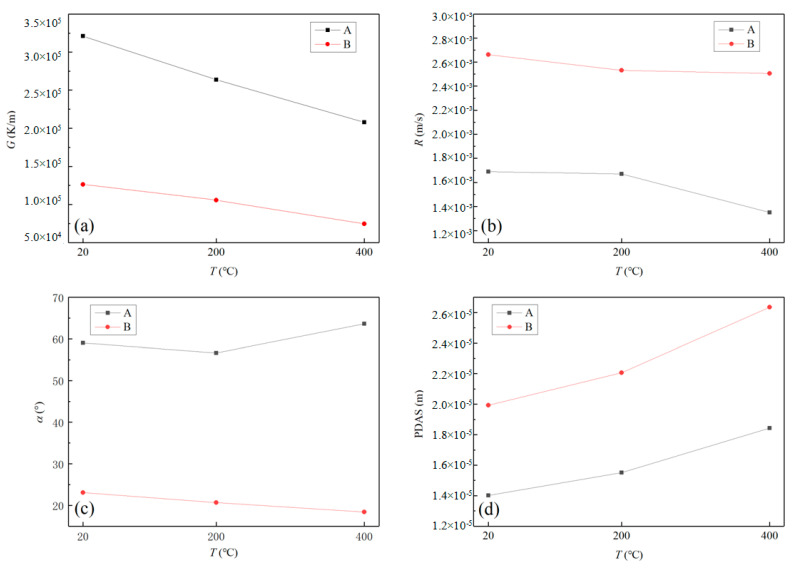
Comparison of parameter values for three preheating conditions of double-layer cladding: (**a**) temperature gradient; (**b**) solidification rate; (**c**) solidification angle; (**d**) PDAS.

Figure 10 displays the parameter values of D (Z = 0 mm) and E (Z = 1 mm) for double-layer cladding under three preheating temperature conditions. When there is no preheating condition, the temperature gradient of the second layer is reduced by 60.7% compared to the first layer, and the solidification speed increases by 57.3% as illustrated in Figure 10a,b. The temperature gradient decreases as the heating effect of the initial layer reduces the temperature difference between the cladding layer and the substrate. As the temperature increases, conduction heat accelerates and leads to a significant rise in the solidification rate. Furthermore, the effect of the *α* angle is greater, causing a decrease of 64.8% as illustrated in Figure 10c and encompassing a 31.8% impact on the increase of PDAS as shown in Figure 10d. Significant differences in the direction of solidification velocity are noticeable at distinct points. Comparing the characteristic values at each point under three preheating conditions, the relationship between them is identical to that of single preheating. With an increase in preheating temperature, *G* and *R* decrease, and PDAS increases consequently.

### 4.2. Flow Field Distribution

**Figure 11:**
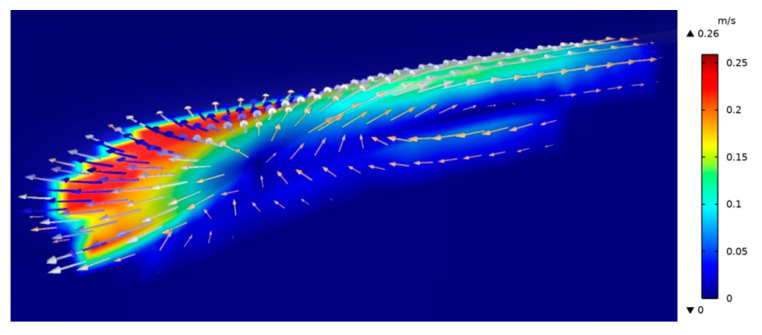
Flow field distribution on the surface of the molten pool.

Figure 11 depicts that the laser induces the liquid metal to flow from the center of the melt pool to its edges because of the high temperature at the center of the melt pool and the low surface tension and this results in the highest surface flow speed at the edges. Simultaneously, some molten metal flows back to the center of the melt pool at the bottom to close the convection zone, resulting in the highest surface flow velocity at the edge. It is important to note that the surface flow layer is much thinner than the depth of the molten pool due to the lower viscosity of liquid metal. This results in the surface shear stress caused by the surface tension gradient of the molten pool being unable to penetrate deep into the molten pool. However, the thin flow layer on the surface effectively transports heat from the center of the molten pool outward. As a result, the highest temperature and depth of the molten pool are significantly lower than when the surface tension is constant. Additionally, a ring convection is formed inside the melt pool around the laser action point, causing the melt pool’s surface to flow from both sides of the laser action point. To counterbalance the solute movement to both sides, the fluid in the center of the melt pool rises. As some fluid flows sideways, it converges toward the center of the melt pool and rises, while the remainder flows along the bottom of the melt pool and ultimately solidifies there. The flow of liquid metal from the center to the edge amplifies heat diffusion within the melt pool, thereby narrowing the temperature gradient and broadening the solid/liquid boundary.

### 4.3. Microstructure Morphology Analysis

**Figure 12:**
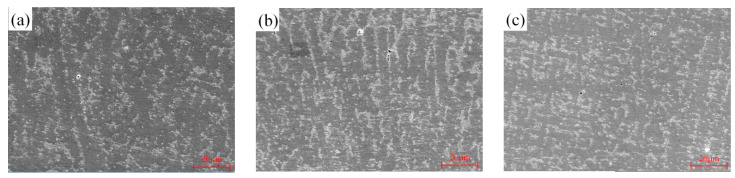
Three preheating SEM images (1000 times): (**a**) no preheating; (**b**) preheating at 200 °C; (**c**) preheating at 400 °C.

Figure 12 displays SEM images of dendrites located at the base of the melt pool in the Inconel 718 sample across three distinct processes. In this research, the substrate was heated up to a temperature that did not surpass the transition temperature (650 °C). As a result, preheating had no influence on the microstructure of the substrate or the creation of carbides in it. Figure 12 demonstrates various microstructures in the deposition zone as a function of diverse preheating temperatures. The dendrite arm spacing morphology changes with preheating temperature variation. As the temperature increases, column arm spacing increases while the volume fraction of the martensite structure decreases. The dendrite morphology and characteristics are directly related to the temperature gradient and solidification speed.

The white Laves phase can be identified as it precipitates irregularly along the dendrites at the bottom of the cladding layer. The Laves phase volume increases as the preheating temperature rises. After analyzing SEM images with binary processing, it was discovered that preheating to 400 °C resulted in a 33.5% higher proportion of Laves compared to the unpreheated state. Due to the low cooling rate during solidification, a greater number of Nb atoms tend to migrate to the interdendritic region, thereby resulting in more Nb-rich areas in the melt pool. Due to the low cooling rate during solidification, a higher concentration of Nb atoms tends to accumulate in the interdendritic region, resulting in an increase in Nb-rich areas in the melt. Additionally, more areas meet the conditions for Laves precipitation, leading to the formation of coarse Laves phases. Furthermore, a higher cooling rate results in the formation of smaller PDAS, which close during secondary arm dendrite growth and separate the remaining liquid into isolated Nb-rich regions, thereby forming dispersed Laves. As the partial pressure of dendrite arm spacing increases, the regions rich in Nb within the remaining liquid often take on continuous geometric shapes, leading to the formation of long-chain Laves phases, which makes them more susceptible to liquefaction cracks. Additionally, as the consumption of Nb element increases, the solid solution effect of alloy elements in austenite weakens, and the mechanical properties of the alloy also deteriorate. Therefore, it is necessary to comprehensively consider the preheating temperature.

## 5. Conclusions

By establishing a numerical model to investigate the effect of Inconel 718 substrate preheating on the process of laser metal deposition, and further analyzing the solidification behavior of the melt pool at varying preheating temperatures in conjunction with the cladding experiment, the following conclusions have been drawn:

(1) As the preheating temperature increases, the temperature gradient significantly decreases, leading to a decrease in solidification speed and cooling speed, while PDAS increases accordingly.
(2) In the case of double-layer cladding, the first layer’s heating effect reduces the temperature difference between the substrate and the cladding layer, resulting in a reduction in the second layer of cladding’s temperature gradient. As temperature increases, heat conduction accelerates, and solidification speed significantly increases, which comprehensively affects the increase in PDAS. Preheating the substrate to 400 °C increases PDAS by 31.8% compared to the unheated state.
(3) As the preheating temperature increases, the dendrite spacing gradually increases, the Laves phase precipitates increase, and it is easier to form coarse long-chain Laves phases. And it was discovered that preheating to 400 °C resulted in a 33.5% higher proportion of Laves compared to the unpreheated state.

## Figures and Tables

**Table 1:** Physical parameters of Inconel 718.

Parameter	Value	Unit
Inconel 718 liquidus, *T_L_*	1647	K
Inconel 718 solidus, *T_S_*	1571	K
Solid density *ρ_m_*	8190	kg/m^3^
Specific heat, *C_p_*	435	J/kg/K
Convective heat transfer coefficient, *h_c_*	10	W/(m^2^·K)
Latent heat, *L_f_*	145	J/kg
Dynamic viscosity, *μ*	0.006	kg/(m·s)
Surface tension coefficient *σ*	1.842	kg/s^2^
Temperature coefficient of surface tension d*σ*/dT	−0.11	kg/(s^2^·K)

**Table 2:** Basic simulation parameters.

Process Parameter	Value	Unit
Laser power, *P*	2000	W
Scanning speed *V_s_*	3	mm/s
Spot radius, *r_l_*	1.1	mm
Powderfeedrate, *m_f_*	10	g/min
Powder flow radius, *r_p_*	2.5	mm
Laser absorption rate, *η_p_*	0.28	1

**Table 3:** Inconel 718 powder element content (wt.%).

Element	Ni	Cr	Fe	Nb	Mo	Al	Co	C
Percentage	52	18.7	18.1	5.16	2.94	0.53	0.1	0.037

## Data Availability

The data presented in this study are available on request from the corresponding author.
